# Oral Prophylaxis Versus Sarcomas

**Published:** 1922-06

**Authors:** M. Rattner


### ORAL PROPHYLAXIS VERSUS SARCOMAS

BY M. RATTNER, D.D.S.

Why should the dental profession now pause in its mad haste to discover new holes for the placing of gold or amalgam fillings and inlays? Is it because the dental world has come to realize what an important part it has come to play in the welfare of the nation? Is it on account of the notoriety that the dental profession has received that it is a panacea for all ills? Or is it on account of the blustering attitude that the dental college now has, because it feels that at last it has come into her own? It is not for any of these reasons that we have put on the "brakes" (to use a slang expression)—but because the world has come to realize the impossibility of curing disease. It has come to the conclusion that the only way that we may eliminate disease is not by the promiscuous use of medicine and the surgeon's knife, but by prevention. This knowledge has developed new methods of treatment to the medical as well as to the dental professions. The dominant idea now is prevention, which is only another word for prophylaxis.

What does this mean to the dental profession? Just this, that when we realize all the jaw sarcomas, and carcinomas, and their dependence upon dental ills and irritations, then we realize that prevention or prophylaxis has not been taught "sincerely and accurately, but abstractly and not concretely." How many of us know that there are over two thousand people dying every year from the fearful ravages of mouth tumors? Think of that. This just proves that the dental profession is guilty of the highest kind of negligence. And why? This is the reason: too often and only too often do we casually notice growths in the mouths—and when we do see them (a large percentage never go that far)—we look upon them as harmless growths to be "snipped off," or to take the policy of "hands off." This condition is due to the fact that the dental colleges do not teach their students to recognize these growths nor to even look for them. They are mostly interested in teaching the students to look for cavities to fill rather than to prevent incipient diseases.

I wonder how many realize that a large percentage of malignant diseases could be prevented by oral hygiene, or oral prophylaxis? By prophylaxis I do not mean the mere brushing of the tooth surfaces with silex or pumice on a brush-wheel, as too many practitioners do, but I want this term to have the full significance of actual prevention, the best methods of every form of prophylaxis. This means the keeping of the mouth not only clean, but the removing of all forms and sources of irritation that may be found in the oral cavity. In this category I should specially mention the removing of all overhanging fillings, jagged root ends, ragged tooth surfaces, rough fillings, etc. This is not a new thought at all—it wasn't for the 20th century to discover that dental irritations will cause tumor formations, but it was Hippocrates, the world's greatest doctor and dentist that made the following assertion: "If there is an ulcer in the mouth look for the broken-down tooth."

The mouth actually ranks first as a place for incipient malignant disease. This is due to the fact that the oral cavity is subject to more chronic irritations than any other part of the body. One does not realize how important the mouth is until something goes wrong. Just to think that all the food that we eat to keep our bodies going comes through that organ; and look at the abuse it suffers. We eat hot and cold foods; most of us use tobacco—not even the ladies are excepted in this—not to mention the rapid manner in which we chew our food.

Out of 638 reported cases of mouth cancers they attributed the causes to neglected teeth. This shows how important it is for every individual to keep his mouth clean and the teeth in good order. Cleanliness and good dentistry should keep the public free from sarcomas and carcinomas.

This sarcoma is a connective tissue form of tumor, having for its most important divisions the giant cell type, round both large and small, spindle cell and mixed round and spindle, alveolar and melanotic, the endothelial and mixed types.

In a general way we may say that in the jaw, sarcomas occur less frequently than carcinomas. They average about two to three. They occur less frequently in the male than in the female. They have a very slight tendency to metastasize, this being the case most often in the round cell type. When they do metastasize they do so through the regional lymphatics and very seldom, if ever, through the viscera. The question of age has met with a great deal of diverse opinions. In a general way we may say that no age is free from sarcomas. The youngest case that I have on record is nine months, and the oldest is eighty-six years old. Out of 452 cases of jaw sarcomas the following data has been gathered:

Ninety cases were found among those whose ages ranged from 11 to 20; 74 from 21 to 30; 76 from 31 to 40; 61 from 41 to 50; 63 from 51 to 60; 36 from 61 to 70; 5 from 71 to 80; 1 at 86; 46 from 0 to 10.

As to the malignancy of the sarcomas, all agree that the round cell is the most malignant and the giant cell type the least. The latter has caused a great deal of trouble, as some authors believe that they should not be classified with sarcomas but with a separate heading under epulis. Here, too, one finds a differentiation as to the meaning of the word epulis.

This is one of the most common tumors that may be found in the mouth. Originally it meant a tumor springing from the gum, but this has caused a great deal of confusion as there is no distinction between benign and malignant. For its histology one may say that they contain many giant cells with a mixture of round and spindle cells. There is considerable blood pigment and polymorphonuclear leucocytes. Microscopically, they are pedunctulated masses and deep red in color.

They occur at any age but are more frequently found during the ages from ten to twenty years. The female sex is more prone to it than the male. They are primarily not malignant but may become so.

In its etiology one would find dental caries a very large factor. Dental hygiene that is poor is another—

irritation that comes from ill-fitting artificial dentures is a third—pipe smoking and continued traumatism is a fourth—and the influence of pregnancy is a fifth factor that one may consider.

The epulis if left alone will become very malignant. It must be removed at the earliest time. This shows how important it is for the dentist to recognize this type of tumor. And in the removal one should first excise all the tissue and then cauterize all the surrounding tissue. It is an established fact that it is far better not to touch an epulis at all than to just partially remove it. There is a very rapid recurrence and a certain deadly malignancy.

Giant cell sarcomas may be endosteal in origin. In the upper jaws they arise in the alveolus or in the facial wall of the antrum where this joins with the alveolar process. In the lower they arise in the alveolus or in the body of the jaw below the level of the teeth. The malignant epulis usually arises in the alveolar margin.

Round, spindle or mixed cell sarcomas may arise from the center of the bone or from under the periosteum. The lower part of the lining membrane of the antrum is the most favorable site.

The round cell sarcomas are very rapid in their proliferation and they fungate to a considerable extent if there has not been a complete removal of the tumor. They may extend in two ways—one along the inferior dental canal, and two, the same extension upwards towards the frontal sinus along the infundibulum—this latter was pointed out by Keen.

The endothelioma originates in the flattened lining of the endothelial lymph spaces or capillary blood vessels. They clinically resemble the spindle cell variety and are histologically epithelial in appearance.

Let us now consider why it is so difficult to ascertain at first glance why a swelling is not at all times a tumor.

A swelling is an enlargement that may be due to many different causes. These enlargements may be classified by Hopewell-Smith into solid and fluid. And then these may be again classified as swellings due to cysts, syphillis, tuberculosis or actinomycosis. Then there are the following diseases that one must consider. Take such cases as leontiasis osii, in which the cranial bones become thickened, show irregular bosses of new bone which may block the nasal fossae, obliterate the antrum and seriously encroach upon the orbit displacing the eyeball and giving rise to deformities. Acromegaly is another disease that may be mistaken for a sarcomatous or carcinomatous growth. It increases the size of the superior and inferior maxillae. The condition called osteitis deformans, too, may be mistaken for a tumor. In this disease the jaws and other bones become enlarged and present a porous appearance.

The means of diagnosis is the one that is of especial interest to the dentist. It is one of the most misused words in the English language. We are all too willing to satisfy the patient that we know exactly the trouble and are but too eager to venture an opinion or a "diagnosis" of the case. How much better the dental profession would be had they been taught that it is far the safest method to wait until they are fully aware of all the conditions before answering as to just what is the trouble. The scientific dentist or doctor is not one who will put his reputation in jeopardy by a so-called snap diagnosis. Today the profession is doing that almost every hour. We are willing to sacrifice our reputations on the merest bit of information. Take for example the X-ray. That, in my opinion, has been both a curse as well as a boon to dentistry as well as medicine. It has been the cause of many an imperfect, unscientific and ignorant diagnosis. At this time I recall to mind the decision of a surgeon in Philadelphia, who decided that a tumor was a giant cell sarcoma just from the evidence submitted by an X-ray. That of course is an impossibility, as all that one may learn from an X-ray is that there is a swelling in the jaw—and NOT that there is a malignant tumor. That of course is impossible—the only means that we have in deciding that is by microscopical examination—that is the last word in the diagnosis of tumor formations.

The treatment of Sarcomas differs very much. The oldest treatment is that of excision. New, of the Mayo clinic, uses radium and cautery. This method has not as yet been established, as it is based on too few successes and these have been calculated upon too short a period of time. The time limit for recurrence should be not months but years, and at that five would be a correct figure. The other means of operation are excision and radium, excision and cautery, excision and Coley's fluid, and the use of radium and X-ray. The latter is very dangerous and its practice should be limited to competent operators. Coley's fluid or serum is made up of prodigiosis and erysipelis. This serum has been used successfully in some inoperable cases.

The symptoms are many and varied. We may group them as follows: Pain, swelling, neuralgia, nasal obstruction, nasal discharge, trismus, displacement of nose, broadening of nose, growth in nasal fossæ visible with the speculum, displacement of eye, impairment of vision and facial paralysis.

To sum up, we may say then that sarcomas occur less frequently than carcinomas; that they occur more frequently in the female and that they are found more often in the superior than in the inferior maxillae; that the giant cell type is the most benign and that the most malignant is the round cell type; that they occur at any age but are found more at the ages between ten and twenty; that the treatment is the surgical one and radium; that radium be limited in its use; that the means of diagnosis should be the pathological report; that the symptoms are many and varied; that there is very little metastasis; that there will surely be a recurrence if the whole tumor is not extirpated; that the time limit for judging whether excision has been a cure or not, be over a period of five years. And furthermore, that dentists be educated to the place where they shall be able to recognize malignant growths and advise their removal, as early excision is an important factor.

The question of jaw sarcomas will be reduced to a minimum when the dental student will be taught to examine the whole mouth correctly and to recognize all that he sees there. Let the student learn to honestly and conscientiously perform dental prophylaxis and the practitioner will not have to answer the question of negligence, for sarcomas of the jaws will then be reduced to a minimum.

BIBLIOGRAPHY

J. C. Bloodgood—Dental Review, May, 1917.
V. A. Latham—A. M. A., May, 1898.
Turner—Brit. J. D. S., April, 1902.
Perthes—Deutsche Chir. Lieferung, 33A.

